# To American Physicians Interested in the Alcoholic Problem

**Published:** 1908-04

**Authors:** 


To American Physicians Interested in the Alcoholic Problem.—During 1907 over two hundred papers, lectures and pamphlets were published in Europe and America concerning alcoholism and inebriety from a purely scientific point of view. Many of the authors complain that these papers were practically lost, because they did not reach medical men interested in the subject. The Scientific Federation Bureau, organized in Boston two years ago for the purpose of collecting and disseminating the facts concerning the alcoholic problem, proposes to secure a list of medical men who are interested in the scientific study of the alcoholic problem. This list will be valuable for authors and students, who wish to address a special audience of physicians, not only to increase their interest, but to stimulate more exact studies of the subject. Such a list will enable the Bureau to extend its work of accumulating papers and reprints of all that is written and keep authors and readers familiar with the work that is done. All physicians who are interested in the scientific study of the alcoholic problem and research work, and the studies of medical men at home and abroad, are urged to send their names and addresses so as to be registered and receive copies of papers and abstracts from authors and from others who may wish to have their papers read by interested persons. As chairman of the board of directors of the Scientific Federation Bureau, I earnestly request all physicians, who would like to know more of this work, to send me, not only their names, but the names of other medical men who would care to keep in touch with the new literature along scientific lines, and the latest conclusions concerning this problem which come from further studies. Address T. D. Crothers, M. D., Chairman, Hartford, Conn.

